# Fast IMRT by increasing the beam number and reducing the number of segments

**DOI:** 10.1186/1748-717X-6-170

**Published:** 2011-12-09

**Authors:** Klaus Bratengeier, Mark B Gainey, Michael Flentje

**Affiliations:** 1 University of Würzburg, Department of Radiation Oncology, Josef-Schneider-Str. 11, 97080 Würzburg, Germany

**Keywords:** IMAT, Step and Shoot IMRT, VMAT, Optimization

## Abstract

**Purpose:**

The purpose of this work is to develop fast deliverable step and shoot IMRT technique. A reduction in the number of segments should theoretically be possible, whilst simultaneously maintaining plan quality, provided that the reduction is accompanied by an increased number of gantry angles. A benefit of this method is that the segment shaping could be performed during gantry motion, thereby reducing the delivery time. The aim was to find classes of such solutions whose plan quality can compete with conventional IMRT.

**Materials/Methods:**

A planning study was performed. Step and shoot IMRT plans were created using direct machine parameter optimization (DMPO) as a reference. DMPO plans were compared to an IMRT variant having only one segment per angle ("2-Step Fast"). 2-Step Fast is based on a geometrical analysis of the topology of the planning target volume (PTV) and the organs at risk (OAR). A prostate/rectum case, spine metastasis/spinal cord, breast/lung and an artificial PTV/OAR combination of the ESTRO-Quasimodo phantom were used for the study. The composite objective value (COV), a quality score, and plan delivery time were compared. The delivery time for the DMPO reference plan and the 2-Step Fast IMRT technique was measured and calculated for two different linacs, a twelve year old Siemens Primus™ ("old" linac) and two Elekta Synergy™ "S" linacs ("new" linacs).

**Results:**

2-Step Fast had comparable or better quality than the reference DMPO plan. The number of segments was smaller than for the reference plan, the number of gantry angles was between 23 and 34. For the modern linac the delivery time was always smaller than that for the reference plan. The calculated (measured) values showed a mean delivery time reduction of 21% (21%) for the new linac, and of 7% (3%) for the old linac compared to the respective DMPO reference plans. For the old linac, the data handling time per beam was the limiting factor for the treatment time reduction.

**Conclusions:**

2-Step Fast plans are suited to reduce the delivery time, especially if the data handling time per beam is short. The plan quality can be retained or even increased for fewer segments provided more gantry angles are used.

## Background

Fast delivery of radiation techniques sparing organs at risk (OAR) is desirable for several reasons. First, some authors favor fast application due to biological effects [1]. Second, short delivery times reduce the problems related to patient movement. Third, more patients can be treated with the same linear accelerator (linac). Intensity modulated radiation therapy (IMRT)[2] and intensity modulated arc therapy (IMAT)[3] were developed to improve OAR sparing without reduction of tumor control [4]. Both methods tend to be time consuming and to increase the total delivery time with respect to conventional conformal radiation therapy (CRT). However, Otto demonstrated that single arc IMAT plans with delivery times of a few minutes are possible (volumetric arc therapy, VMAT)[5]. Many researchers focus on investigating and improving arc based techniques. In contrast, IMRT tended to become a reference technique that provides the gold standard for the plan quality but does not leave a lot of room for improvement. However, many hospitals are currently not equipped with linacs capable of IMAT delivery. Therefore a reduction of delivery times without compromising plan quality using less demanding IMRT-capable linacs could be useful [6].

### Lower delivery time and better plan quality using fewer segments and more beams

**Figure 1:**
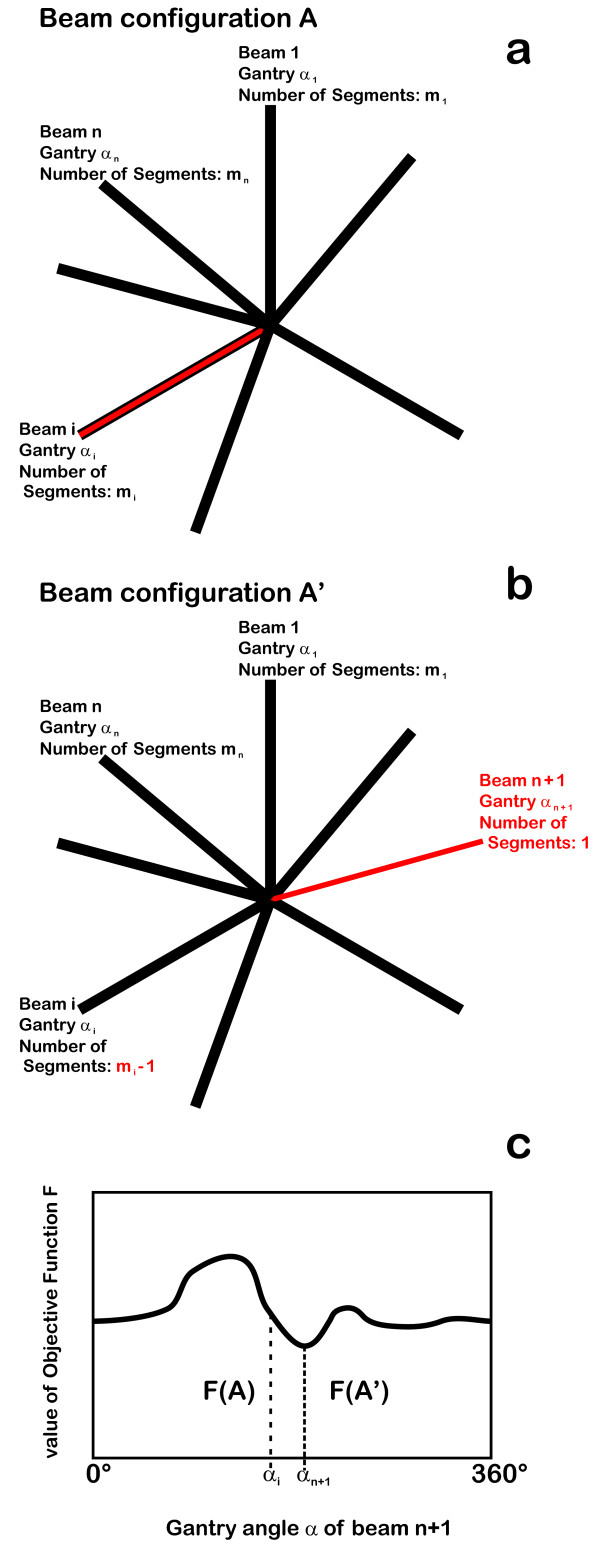
**More beams and less segments could result in better plans**. Plan changes from A to A': the number of segments and gantry angles are the same for all beams except beam i. Beam i: number of segments is reduced by 1, beam n+1 - containing only one segment - is inserted at gantry angle α_n+1_. Segment shapes and weights of all beams are newly optimized, a better plan is found. After certain number of repetitions, only one segment per beam remains. Finally some beams can be dropped, even if this decreases the quality of the plan, as long as the plan does not become worse than the start plan.

Reduction of the number of segments can potentially reduce the delivery time, as long as the total monitor units do not increase. This reduction should be enabled by increasing the number of gantry angles. A thought experiment can demonstrate this. Let A (*α_1_, ... α_i_, ... α_n_*) be an *n*-beam, *N*-segment IMRT plan of acceptable quality, corresponding to a local minimum of the objective function *F*. Let *m_i _*> 1 be the number of segments at the angle *α_i _*(Figure 1a). Let us move one segment from a beam at angle *α_i _*to a new beam at a new gantry angle α_n+1 _which can be chosen freely. Then the total number *N *of segments is constant: *N*(A) = *N*(A') (Figure 1b).

Now the optimization process is initiated and a new minimum *F'*(*α_n+1_*) is found, depending on the chosen gantry angle *α_n+1 _*(Figure 1c). For a process that is able to find the global minimum, a non-empty set exists such that { *α_n+1 _*| *F'(α_n+1_) *≤ *F *} ≠ {}. That means that there is always a possibility to increase plan quality by distributing the same number of segments over a wider range of gantry angles unless A was the best possible solution for all possible gantry combinations which is extremely improbable. After (*N - n*) inductive steps, we obtain a plan with *N *beams at *N *different gantry angles with one segment per beam and plan quality *F' ^N ^*<*F*. This increase in quality can be invested in a solution B with fewer segments: *N*(B) <*N*(A). Our aim is to find a solution with *F' *^*N*(B) ^≈ *F*.

These general considerations do not provide a practical solution. Rather than trying to determine the best beam orientation for a limited number of beams, in this case the problem is to find the segment shapes for an extended number of single-segment beams. This problem resembles the VMAT optimization problem [5]. Thus, similar to VMAT-like methods, at the first step a limited number of IMRT beams could be created, then the segments could be re-distributed over a certain range of gantry angles and then be further optimized [7]. Another method could be based on the direct aperture optimization [8], including simulated annealing [9], beginning with single segment beams [10,11].

We base our search for a single segment beam solution on the 2-Step IMRT technique which analyses the topology of the planning target volume (PTV) and organs at risk (OAR). Only few groups have published techniques [12-15] that take account of the blocking of the PTV areas nearby the OAR in a similar way. Such considerations lead to additional segments similar to the "Brahme-peak", an increase of the fluence for the PTV areas proximate to the OAR [4]. 2-Step IMRT demonstrated its capability in comparison with direct aperture optimization-based algorithms for step and shoot techniques [16] and can be used as a basis for the ad hoc adaptation of IMRT plans to the daily target [17].

The purpose of the study is to develop and evaluate 2-Step based fast deliverable IMRT plans of quality comparable or better then the conventional IMRT plans provided by commercially available software. We propose the 2-Step Fast method which uses more beams than is typical for IMRT, each beam having only one segment. This allows the multi-leaf-collimator (MLC) to adjust the leaves during gantry motion between the beams.

## Materials and methods

This work is closely related to the study of fast deliverable IMAT methods that also use the 2-Step IMRT technique and which were compared to VMAT plans [18]. The same study design was chosen as in [18], the same patient models, dose prescriptions, IMRT reference plans, sets of optimization objectives and methods of quantifying the plan quality. Therefore the results can be compared directly.

The Philips Pinnacle3™ therapy planning system (TPS) (Philips Radiation Oncology Systems, Fitchburg, WI, USA) which includes the direct machine parameter optimizer (DMPO^®^) was used for all calculations of dose distributions.

To avoid effects of the special choice of planning conditions, some parameters were varied: The clinical version 8.0 and the pre-clinical version 8.9 were both applied for the reference plans. A twelve year old linac Siemens Primus™ ("P10") (Siemens Healthcare, Erlangen, Germany) with IM-MAXX™ technology ("old" linac), a five year old Elekta Synergy™ "S" (Elekta AB, Stockholm, Sweden) linac with 4 mm leaves (Beam Modulator™, "S4") and Elekta model Synergy™ ("S10") with 10 mm leaves, ("new" linacs) have been commissioned in the TPS. To compare the output of the linac adequately, effective Monitor Units were defined: 100 MU_eff _are needed to deposit 1 Gy using a 100 × 100 mm^2 ^-field at 100 mm depth for SAD 1000 mm. All plans had two consecutive optimization runs 40 steps each, making up a total of 80 optimization iterations.

Objective values were defined as weighted quadratic penalties. Plans were optimized by minimizing the composite objective value (COV) [19], which is the weighted sum of all objective values. For the patient PTVs two objectives described the requirements near the minimal dose (e.g. D_100 _≡ D_min _> 95% and D_98 _> 97% of the aimed PTV dose) and two objectives limited the dose to the high dose region (e.g. D_02 _< 102% and D_00 _≡ D_max _< 104%). Two further objectives limited the minimum and maximum dose of the central plateau of each PTV to avoid cold and hot spots in the PTV. For the OARs proximate to the PTV, up to three objective values defined the desired course of the DVH. No hard constraints were set for the optimization, rather, objectives were appropriately weighted, and the weights were chosen in a wide range between 0.1 and 100. If after 40 optimization steps the segment or beam had less than 2 monitor units (MU), it was discarded.

The patient models and methods of plan quality characterization are described in the appendix.

### DMPO 9 reference plan

The results of 2-Step Fast method were compared with a step and shoot IMRT reference plan which consisted of 9 equidistant beams (DMPO 9). The IMRT planning was performed using the commercial DMPO^® ^optimization algorithm [8]. The objectives developed for the reference plan were also applied for all other plan types.

### 2-Step IMRT algorithm with successive fine tuning

**Figure 2:**
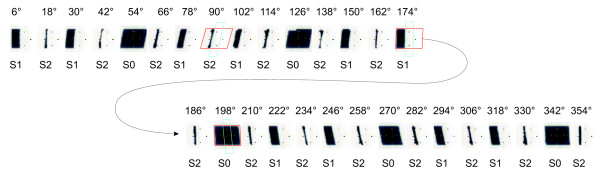
Segments of 2-Step Fast 30*. 2-Step Fast 30* segments for the Quasimodo phantom. Upper row: gantry angles. Lower row: Segment type. For three S0, S1, S2 segments the projection of PTV (red contour) and OAR (green contour) is shown.

The 2-Step IMRT technique uses the analysis of the PTV/OAR geometry [18]. It generates up to three types of segments per gantry angle in order to obtain approximately homogeneous dose distribution for a concave PTV around the OAR [20,21]:

• Optional zeroth order segments (S0) cover the whole PTV without consideration of the OARs. Such PTV-conformal segments smooth the dose distribution but increase the dose load to the OAR. For best OAR sparing in the case of concave targets surrounding the OAR these segments must be omitted.

• First order segments (S1) cover the PTV but block out the OAR. These segments spare the OAR but create some underdosage in the areas of PTV adjacent to the OAR (see Figure 2). Up to this point, the method is similar to many others that simply spare the OAR.

• Second order segments (S2) saturate underdosed PTV areas adjacent to the OAR that remain from the first order segments. S2 segments are generally narrow, directed only to the PTV in the circumference of the OAR, whilst sparing the OAR [22] (see Figure 2). Second order segments can be regarded as approximation of the "Brahme-peak" [4], the sharp fluence increase in the immediate vicinity of the OAR. Second order segments form a second fluence step on top of the conformal and first order segments. They were demonstrated to be very important for the cases requiring a steep dose gradient from the OAR to the (partially) surrounding PTV [22]. In this manner a more homogenous dose distribution in the PTV without trade-offs in OAR sparing can be achieved.

In the second planning phase, the shapes and weights of the pre-defined segments were fine-tuned [16,18]. For the pre-segmentation only the dominating OAR was considered in addition to the PTVs. However, for the fine tuning of segment shapes and weights other OARs were also taken into account according to their objective values. The fine-tuning was performed by the DMPO^® ^optimization engine. The DMPO^® ^engine consists of three modules: the first module performs fluence optimization independent of machine parameters; the second sequences segments and the third optimizes shapes and weights (MUs) of the generated segments taking machine parameters into account. For the 2-Step based plans, the predefined segments were fed into the third module of the DMPO^® ^optimizer. The optimizer was configured to vary segment weights and apertures only.

### Standard 2-Step IMRT plan

The 2-Step IMRT plan [16] with the same number of beams (9) and same gantry angles as in the reference plan was generated ("2-Step IMRT 9"). 2-Step IMRT 9 compared with DMPO 9 shows the effect of special 2-Step segment generation scheme. Additionally, plans with 15 equidistant beams ("2-Step IMRT 15") were created to investigate the effect of additional gantry angles and segments.

### 2-Step-Fast and 2-Step Fast* plans

We propose fast versions of 2-Step IMRT technique: 2-Step Fast and 2-Step Fast* IMRT, which

1) use the gantry positioning time for positioning the leaves;

2) reduce the number of segments such that fewer segments distributed over more gantry angles result in clinically acceptable dose distributions.

Only one segment type (S2, S1, or S0) was utilized per gantry angle, but more equidistant gantry angles were used than in the standard 2-Step IMRT.

To enable extraordinary sparing of the OAR, 2-Step Fast integrated only segments of the first and second order should be used. These types are not directed to the OAR. The segment orders alternated from one gantry angle to the next according to the scheme S1 S2 S1 S2 S1 S2... (in short: {S1 S2}^m^). 2-Step Fast was used to spare the lung in the breast case, the spinal cord in the spine case and the OAR in the Quasimodo case.

2-Step Fast* contained segments of all orders, allowing smaller number of monitor units. The sequence {S0 S1 S2}^m ^was used for the prostate case, {S0 S2 S1 S2 S1 S2}^m' ^for the Quasimodo case (see Figure 2).

For fast delivery, the leaf travel between the segments should be as short as possible. Therefore, only left-sided segments S1 and S2 were chosen with respect to the dominant organ at risk. If no valid S1 or S2 segment existed for the given gantry angle, the beam was discarded.

Additionally, DMPO^® ^plans with a single segment per gantry angle were generated (DMPO Fast) for the same gantry angles as for 2-Step Fast or 2-Step Fast*. DMPO Fast was established to explore the effect of predefined segments as provided by 2-Step Fast.

### Plan delivery time *T*

The delivery time was measured for 12 plans and estimated using a simple formula for 28 plans. The plan delivery time *T *depends on

1) the number of equidistant gantry angles *n*;

2) the number of segments *N*;

3) the gantry rotation time between beams *τ_GSS_*. The gantry rotation time includes the start-stop time of the gantry *τ_SS_*, and the gantry rotation speed per degree *v_G_*:

(1) $$\tau_{GSS}=\left(\tau_{SS}+\frac{360^{\circ }}{v_{G}}\right);$$

4) the segment shaping time, which depends strongly on the leaf speed and the changes in the segment shapes between segments. For the sake of simplicity the mean segment shaping time, ${\bar{\tau }}_{S}$, was considered. Segment shaping and gantry positioning are simultaneous, so the more time consuming process dominates;

5) the pure irradiation time *MU*/, where *MU *is the total number of monitor units,  is the dose rate [MU/min];

6) the data handling time per beam *τ_F_*:

(2) $$T\approx \left(n-1\right)\cdot Max\left\{\tau_{GSS},{\bar{\tau }}_{S}\right\}+\left(N-n+1\right)\cdot {\bar{\tau }}_{S}+\frac{MU}{Ḋ}+\left(n-1\right)\cdot \tau_{F}$$

*T *was estimated using measured parameters at the new linac (old linac in brackets):

*v_G _*= 60 s/360° (78 s/360°), *τ_SS _*= 3 s (3 s), ${\bar{\tau }}_{S}$ = 7 s (12 s), *τ_F _*= 3 s (9 s) for the new and the old linac respectively (Table 1). The discrepancy between measured and calculated time did not exceed 0.6 min.

**Table 1:** Quality parameters *COV *and *S_D_*

Technique	MU_eff_	Field	Segment	norm. COV	S_D_/n	T (old)	T (new)	MU_eff_	field	Segment	norm. COV	S_D_/n	T (old)	T (new)	MU_eff_	field	Segment	norm. COV	S_D_/n	T (old)	T (new)
		**#**	**#**			**[min]**	**[min]**		**#**	**#**			**[min]**	**[min]**		**#**	**#**			**[min]**	**[min]**

	Patient Cases																				

	Prostate							Spine							Breast						

DMPO 9	591	9	50	1.00	0	11.8	7.8 [7.4]	888	9	50	1.00	0	12.3	8.2 [8.8]	821	10	38	1.00	0	10.9 [10.4]	6.5

2-Step IMRT 9	466	9	38	1.02	0.1	10.2	6.1	755	9	44	1.05	0.4	11.9	7.2	898	10	30	1.03	0.8	10.1	6.2

2-Step IMRT 15	492	15	47	0.53	0	12.9	7.2	711	15	64	1.00	0.2	17.2	9.7	927	15	41	0.69	0.3	13.2	7.5

2-Step Fast								901	34	34	1.07	0.3	14.4	7.5 [7.1]	672	25	25	0.81	0.7	10.3 [10.5]	5.3

2-Step Fast*	468	23	23	0.73	0	9.3	4.7 [5.0]														

DMPO Fast	378	23	23	1.31	0.8	5.7	4.5	565	36	36	1.46	1.1	8.9	7.1	377	25	25	3.49	5.3	9.7	4.9

	Phantom Case																				

	Quasimodo S4							Quasimodo S10							Quasimodo P10						

DMPO 9	525	9	54	1.00	0.9	13.6	8.1 [8.0]	501	9	54	1.00	1.0	13.5	8.1	493	9	54	1.00	1.5	13.4 [13.3]	8.1

2-Step IMRT 9	507	9	40	1.01	0.9	13.6	8.1	526	9	45	0.76	0.8	11.8	7.1	508	9	40	0.82	1.0	10.7	6.5

2-Step IMRT 15	511	15	75	0.48	0	18.6	10.5	538	15	73	0.36	0	18.3	10.3	520	15	67	0.34	0	17.0	9.6

2-Step Fast	802	30	30	1.10	0.6	12.8	6.7 [6.6]	863	30	30	1.02	1.0	13.0	6.8	816	30	30	0.59	0.8	12.9 [12.9]	6.7

2-Step Fast*	504	30	30	1.14	0.6	11.8	6.0 [6.0]	581	30	30	0.74	0.7	12.0	6.1	554	30	30	0.54	0.5	12.1 [12.2]	6.2

DMPO Fast	401	30	30	9.1	6.0	11.5	5.7	397	30	30	21.3	7.9	11.5	5.7	393	30	30	28.2	8.0	11.5	5.7

Overview of the resulting quality parameters COV and SD of all plans used in the study (lower values correspond to better plans). *n*: number of objectives used to calculate the normalized quality index *S_D _/n*. For prostate, spine, and breast cases, the reference achieves *S_D _*= 0 by definition. T: delivery time calculated using Eq. (1), for an old linac with slow data handling and a new linac with fast data handling. Values in brackets: measured values.

S4: Elekta Synergy™ with 4 mm leaves (BeamModulator™), S10: Elekta Synergy™ with 10 mm leaves. P10: Siemens Primus™ with 10 mm leaves. The techniques are presented in detail in the text.

### Quantification of the plan quality

The plans were compared by means of

1. the composite objective function at the end of the optimization process (COV) normalized to COV of the reference DMPO 9 plan. Lower COV corresponds to better plan. The COV is the sum of all weighted objective values and is used as objective function. The set of all objectives reflects the course of the DVHs of all relevant organs and PTVs. Only COV differences of +25%/-20% can be considered as relevant [18].

2. The quality score S_D _per number of objectives, S_D_/n. The quality score S_D _was introduced in [23] as follows:

(3) $$S_{D}=\underset{j}{\sum }\begin{array}{c} \left|M_{j}-C_{j}\right|\\ 0 \end{array}\begin{array}{c} \text{if objective is violated},\\ \text{otherwise}, \end{array}$$

where *C_j _*is the dose objective, *M_j _*is the corresponding plan value. Lower S_*D *_corresponds to better plan. For a plan which fulfills all objectives S_D _= 0. As S_D _for the other studies is refined to the DMPO 9 reference plan, in contrast to the COV, only the "worse" values (unfulfilled objectives) contribute to S_D_, whereas "better" values do not contribute. Therefore for no plan, S_D _could be lower than zero, the value of the DMPO reference plan. S_D _allows comparing the plans among each other except the reference plan. Ameliorations with respect to the reference plan remain undiscovered. Table 1 shows all objectives of all cases. S_D _is divided by the number n of the objectives that were considered.

## Results

### Plan quality

**Figure 3:**
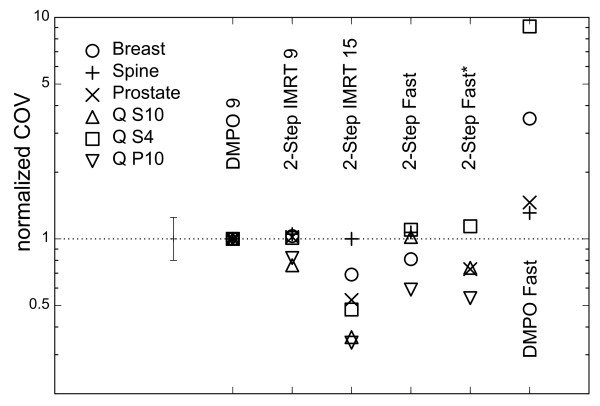
**Plan quality**. The plan quality for all cases and all plan types as reflected by the COV (lower values correspond to better plan quality). The error bar indicates the maximum deviation of COV for all DMPO 9-plans.Two values for DMPO Fast (S10, P10) lie above the presented area (see Table 1).

**Figure 4:**
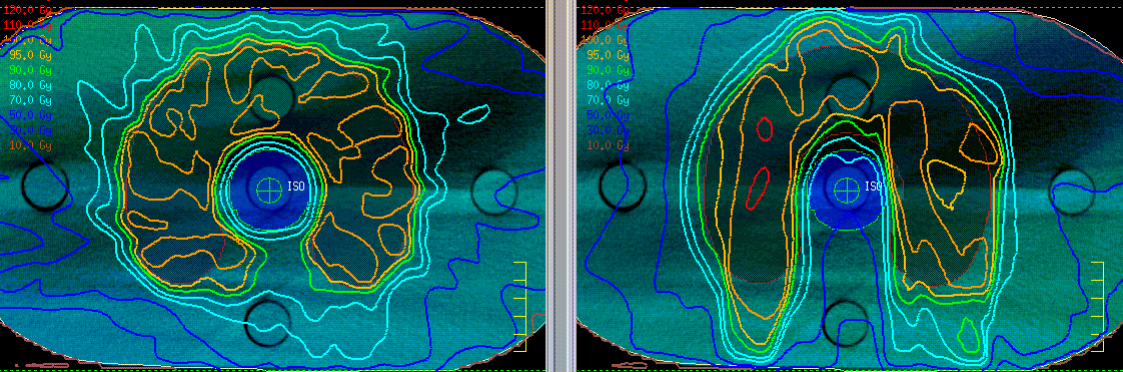
**Dose distribution for a 2-Step Fast and DMPO Fast plans**. Dose distribution of the 2-Step Fast 30 plan (left) and the DMPO Fast 30 plan (right) for the Quasimodo S10 case: axial plane near the centre of the phantom. Isodoses: dark orange: 100%, light orange: 95%, green: 90%, light blue: 80%, 70%, dark blue: 50%, 30%.

The COV of all plans are compared on Figure 3 and Table 1, the S_D_/n in Table 1. As can be seen from Table 1, the quality of 2-Step Fast/Fast* based plans was at least as good, or better than, the reference DMPO 9 plan. The segmentation as starting point for the DMPO optimization is essential: DMPO Fast plans - generated conventionally with restriction of one segment per beam - always produced plans of poorest quality. Figure 4 compares dose distributions for 2-Step Fast and DMPO Fast, showing over- and underdosage in the PTV and hot spots in the healthy tissue in the case of DMPO Fast.

The direct comparison of DMPO 9 and 2-Step IMRT 9 shows no relevant discrepancies, neither for COV nor for S_D_/n. That means that the primary 2-Step segment shaping is equivalent with conventional IMRT if an adequate number of segments are supplied. An increase to 15 gantry angles and a proportional increase of the number of segments lead to better plans in almost all cases. For the spine case, all plans except DMPO Fast had comparable COVs, perhaps due to the moderate requirements.

**Figure 5:**
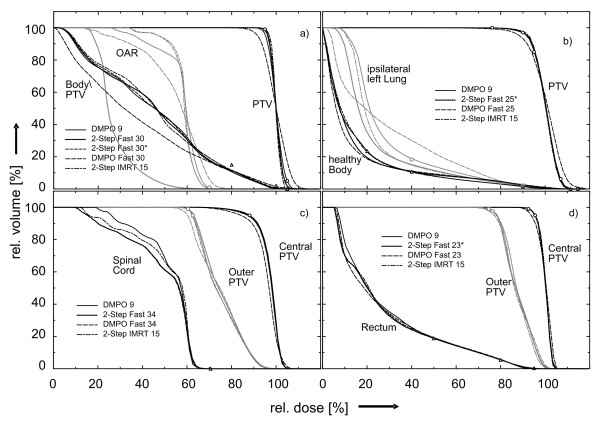
**DVHs for all cases**. DVHs for the plans: DMPO reference plan, 2-Step Fast or 2-Step Fast*, DMPO Fast and 2-Step IMRT 15 for three selected volumes. (DMPO 9 and 2-StepIMRT 9 are very similar). a) Quasimodo (only S10 collimator); b) breast case, c) spine case; d) prostate case. In the case (a) for the 2-Step Fast-technique, the OAR is much more spared than required, because no segment needs to be directed into this volume. A related relative dose distribution is shown in Figure 4. Symbols indicate some objectives used for the calculation of the quality index S_D_: the circle is related to: (central) PTV (a-d); square: OAR (a), ipsilateral left lung (b), outer PTV (c,d); triangle: Body/PTV (a), Healthy body (b), Spinal Cord (c), rectum (d), respectively. In contrast to 2-Step Fast, 2-Step Fast* can include S0-segments with primary radiation also to the OAR.

Figure 5 shows DVHs for all plans. The better OAR sparing of the 2-Step Fast technique (S1 and S2 segments only) with respect to 2-Step Fast* (including PTV-conformal S0 segments) is clearly seen. The difference is not reflected in COV and S_D_, because such low doses to the center of the OAR were not demanded in the set of objectives.

### Delivery time *T*

The calculated delivery times for twelve example plans are also shown in Table 1 (measured times are in brackets). The values of *T *estimated from Eq. (2) agree with the measurements within ± 0.6 min. The delivery time differed between old and new linacs. For the new linac, the calculated (measured) values showed a mean delivery time reduction of -21% (-21%) for the new linacs, for the old it amounted to only -7% (-3%). On the new linac, the 2-Step Fast achieved the reduction of delivery time between -1.4 and -1.7 min, with the average reduction of -17%. The 2-Step Fast* plans achieved the reduction between -1.9 and -2.4 min, with the average of -26%. On the old linac the change in delivery time for 2-Step Fast was from -1.5 to +2.1 min, amounting to insignificant -1% reduction. For 2-Step Fast* plans the reduction from -0.5 to -2.5 min amounted to average -13% reduction. In the case of the old linac the long data handling time *τ_F _*partially outweighs the time saving of concurrent segment shaping and gantry positioning (see Discussion section).

## Discussion

DMPO 9 and 2-Step IMRT 9 plans with slightly fewer segments were nearly equivalent for all cases, as in the earlier work [16]. An increase in the number of gantry angles to 15 combined with increased number of segments clearly improved the plan quality. Further increase of the number of gantry angles combined with a reduction of the number of segments to at least one per gantry angle resulted in plans equivalent to or better than DMPO 9. These findings support the theoretical considerations of the introduction section: more gantry angles enable fewer segments. This conclusion contradicts intuition, perhaps because of experience using current commercially available sequencing algorithms. Using Pinnacle3™ beam segmentation to create single segment beams led to plans of poorest quality (DMPO Fast, see Table 1). Presently, sequencing algorithms do not differentiate between segment orders. As long as the segment sequencing is independent of adjacent beams, the algorithm favors the segments of the same order, though all higher orders (S1, S2) were needed. 2-Step Fast/Fast*, however, explicitly uses the segments of alternating order in the neighboring beams. A similar problem can occur in VMAT sequencing algorithms. There, the second order segments are not adequately represented under certain circumstances, and the plan quality of single arc VMAT decreases [18].

As Table 1 shows, the 2-Step Fast or 2-Step Fast* plans were delivered faster then conventional DMPO 9 plans on new linacs with fast leaf motion and "en bloc" data transfer of the control point sequence for the entire fraction. The delivery time lies between that of a 2-arc VMAT plan [18] and the DMPO plan and can be considered a "poor man's single arc VMAT".

On the old linac, the delivery time of 2-Step Fast or 2-Step Fast* plans did not differ significantly from that of DMPO 9 plans. This is explained by the fact that in old linacs data transfer and data control repetitively take place for every beam, which counteracts a decrease in beam delivery time. For the old linac, with reduced mean data handling time per field of about 4 seconds, the delivery time could also be reduced by 25%. Better hardware-software combinations with *v_G _*= 60 s/360°, *τ_SS _*= 1 s, ${\bar{\tau }}_{S}$ = 5 s, *τ_F _*= 2 s would even allow delivery times of about 4 minutes, comparable to the delivery time of a dual arc VMAT, which according to our experience is necessary for the Quasimodo case.

The gantry start and stop time could also be reduced, if the linac is allowed to irradiate even if small deviations of the gantry angle (e.g. 1°) remain at the beginning of the segment delivery. Then the plan delivery time could be further reduced by about a half up to one minute. This solution approximates single arc VMAT more closely, as it was predicted by Bortfeld [24].

### Outlook

Further work will investigate the application of fast 2-Step based techniques to problems with multiple dominating OARs with strict requirements on tissue sparing, like head and neck cases (with OARs spine, neck parotid glands). The automated generation of the segments of zeroth, first, and second order is currently work in progress. This will enable more comprehensive planning studies to be performed in the future.

2-Step based techniques are well suited for the adaptation problems [21]. Adaptation should also be applicable for 2-Step Fast and 2-Step Fast*.

## Conclusions

2-Step Fast or 2-Step Fast* techniques could serve as an alternative to conventional IMRT plans delivered by commercially available software, if

1) dynamic techniques cannot be applied due to linac limitations, and

2) fast data handling software is performed

2-Step Fast/Fast* generate plans at least as good as DMPO 9. Using 2-Step Fast/Fast* techniques on linacs with "en bloc" data transfer we observed a delivery time reduction of 21%. This provides a solution for clinics with older equipment to perform IMRT much faster and still achieve decent plan quality.

## Appendix

### Cases (patient models)

Three typical clinical cases with one dominant OAR were used as examples in addition to the ESTRO Quasimodo phantom case [23]. A set of "Quasimodo objectives" had been provided by the authors of that study. The parameter set used to achieve these "Quasimodo objectives" consisted of 14 objective values for the Pinnacle3™ optimizer: for the PTV, the central OAR, an envelope around the PTV, the healthy tissue and a help contour in the "neck" of the phantom. The Quasimodo case, although including only one OAR, can be regarded as difficult because of the large OAR radius, the small distance between PTV and OAR and - therefore - stringent demands on the dose gradient, combined with an almost complete exclusion of the OAR.

A prostate case was planned following the rules described in Guckenberger et al. [25,26]: An inner, central PTV, the boost volume, was defined by a 5 mm margin around the prostate and the base of the seminal vesicles, avoiding the rectum. It was surrounded by an outer PTV, defined by a 10 mm margin around prostate and seminal vesicles, with a reduced 7 mm margin towards the rectum. Both were simultaneously irradiated (SIB [27]). 21 objective values were used for the PTV and its integrated boost volume, an envelope around the PTV, a volume containing the seminal vesicles, the rectum within and outside the PTV, and the surrounding healthy tissue.

For the spine case, two dose levels were assigned to two volumes, one encompassing the other and creating a SIB constellation. 28 objective values were applied for the GTV, the central PTV (integrated boost), the outer PTV, the central plateau of the outer PTV, the spine, the esophagus, two envelopes around the outer PTV and the remaining healthy tissue. This case could be considered as the one with the lowest requirements to the concavity of the isodoses in the high-gradient dose region.

The breast patient model (see Figure 1b) was provided by Fogliata et al. [28]. All prescriptions and objectives were adopted from their work. 25 objective values were set for the PTV and its central plateau, the left lung, the right lung, the heart, the contralateral breast, two envelopes around the PTV and the surrounding healthy tissue.

## List of abbreviations

COV: composite objective value; CRT: conformal radiation therapy; DMPO: direct machine parameter optimization; DVH: dose-volume-histogram; IMAT: intensity modulated arc therapy; IMRT: intensity modulated radiation therapy; MLC: multi-leaf-collimator; MU: monitor unit; OAR: organ at risk; PTV: planning target volume; TPS: therapy planning system; VMAT: volumetric arc therapy.

## Competing interests

The authors declare that they have no competing interests.

## Authors' contributions

KB was responsible for the primary concept and the design of the study; he performed most of the calculations and drafted the original manuscript. MG revised the manuscript. MF was responsible for the patients, reviewed patient data and revised the manuscript. All authors read and approved the final manuscript.
